# Zoonotic malaria in Southeast Asia’s changing landscapes: vector complexity, challenges, and future strategies

**DOI:** 10.1186/s13071-026-07485-z

**Published:** 2026-06-06

**Authors:** Nantha Kumar Jeyaprakasam, Abdul Hafiz Zulhaimi, Koon Weng Lau, Sandthya Pramasivan, Wei Kit Phang, Alongkot Ponlawat, Yudthana Samung, Daibin Zhong, Zulkarnain Md Idris, Jetsumon Sattabongkot

**Affiliations:** 1 Biomedical Science Programme, Center for Toxicology and Health Risk Studies, Faculty of Health Sciences, Universiti Kebangsaan Malaysia, Kuala Lumpur, Malaysia; 2 Mahidol Vivax Research Unit (MVRU), Centre of Excellence for Malaria, Faculty of Tropical Medicine, Mahidol University, Bangkok, Thailand; 3 Department of Parasitology, Faculty of Medicine, Universiti Malaya, Kuala Lumpur, Malaysia; 4 Department of Molecular Tropical Medicine and Genetics, Faculty of Tropical Medicine, Mahidol University, Bangkok, Thailand; 5 Vector Biology and Control Section, Department of Entomology, WRAIR-AFRIMS, Bangkok, Thailand; 6 Department of Medical Entomology, Faculty of Tropical Medicine, Mahidol University, Bangkok, Thailand; 7 Department of Population Health and Disease Prevention, University of California, Irvine, CA USA; 8 Department of Parasitology and Medical Entomology, Faculty of Medicine, Universiti Kebangsaan Malaysia, Kuala Lumpur, Malaysia

## Abstract

Zoonotic malaria, particularly *Plasmodium knowlesi*, has emerged as a growing public health concern across Southeast Asia, with increasing incidence reported in multiple countries. The recent detection of additional zoonotic *Plasmodium* species further complicates the epidemiological landscape, underscoring the expanding interface among humans, wildlife reservoirs, and mosquito vectors. Despite this trend, progress towards effective control and elimination remains limited, largely due to substantial gaps in entomological knowledge across many endemic regions. Currently, only a narrow range of vector control approaches is available, and these strategies are often ill-suited to the ecological and behavioural characteristics of zoonotic malaria transmission. A major challenge lies in the complexity of vector species assemblages in Southeast Asia. Some of the vectors belong to species complexes with high morphological similarity, making accurate taxonomic identification difficult. Misidentification of mosquito species can distort assessments of vector competence, biting behaviour, and ecological niche, leading to inaccurate inferences about transmission dynamics and potentially resulting in ineffective surveillance and misdirected vector control interventions. Compounding this issue are rapid landscape changes driven by deforestation, agricultural expansion, and habitat fragmentation, which alter mosquito bionomics. Growing evidence suggests that such environmental disturbances may promote outdoor and forest-associated biting, enhance vector adaptability, and shift transmission risk toward human settlements. Thus, this review synthesises current knowledge on the ecological, behavioural, and taxonomic complexities of zoonotic malaria vectors within a rapidly changing landscape. By critically examining challenges in vector identification, surveillance, and control, we highlight key gaps that impede effective intervention. Finally, we discuss pathways forward, emphasising the need for integrated, context-specific, and One Health-oriented strategies to address the unique challenges posed by zoonotic malaria transmission in Southeast Asia.

**Graphical Abstract:**

**Figure:**
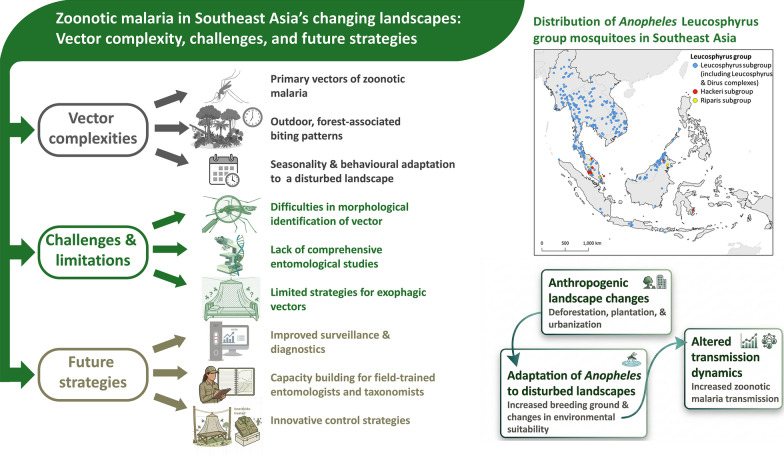

**Supplementary Information:**

The online version contains supplementary material available at 10.1186/s13071-026-07485-z.

## Background

Malaria remains one of the most widespread vector-borne diseases, endemic in many tropical and subtropical countries. Globally, 282 million malaria cases were reported in 2024, an increase of 9 million cases from 2023 [1]. Of these, an estimated 265 million cases (94%) occurred in the World Health Organization (WHO) African Region. In contrast, < 1% of cases were from the WHO Southeast Asia Region, which includes Indonesia, Myanmar, Thailand, and Timor-Leste, and < 1% of cases from the WHO Western Pacific Region covering countries such as Brunei, Cambodia, Lao PDR, Malaysia, Philippines, Singapore, and Vietnam [1].

In Southeast Asia, human malaria cases have decreased over the past few decades [1]. However, in some nations, the incidence of zoonotic malaria, particularly infections caused by *Plasmodium knowlesi* has been increasing [2]. Among the 11 Southeast Asian countries, Timor-Leste has not reported any *P. knowlesi* cases to date, whereas Malaysia has documented the highest burden, accounting for 89% of the 2164 cases reported globally in 2024 [1]. However, the apparent heterogeneity in reported *P. knowlesi* malaria incidence across Southeast Asia likely reflects not only true epidemiological differences but also substantial variation in national surveillance intensity, molecular diagnostic capacity, and integration of zoonotic malaria into routine reporting systems. Since the first large-scale human infection was reported in Sarawak, Malaysian Borneo in 2004 [3], *P. knowlesi* has emerged as the predominant cause of malaria in Malaysia [4]. This zoonotic malaria is primarily transmitted by mosquitoes of the Leucosphyrus Group. Interestingly, the epidemiology of *P. knowlesi* malaria in Southeast Asia closely reflects the geographical overlap between these efficient forest-dwelling mosquito vectors and their natural macaque hosts, *Macaca fascicularis* (the long-tailed macaque) and *Macaca nemestrina* (the pig-tailed macaque) [5]. This convergence of vector and host distribution underpins the high burden of *P. knowlesi* cases reported across several Southeast Asian nations, particularly in areas where human activities encroach upon macaque habitats [2]. Furthermore, naturally occurring human infections with other zoonotic malaria species, such as *Plasmodium cynomolgi* [6] and *P. inui* [7–9], further complicate efforts to eradicate malaria in Southeast Asia [10].

Mass deforestation for agricultural expansion, plantation development, and infrastructure projects, combined with the establishment of human settlements along forest fringes, has significantly contributed to the rise in *P. knowlesi* malaria cases and the emergence of other zoonotic malaria infections [11]. Such anthropogenic landscape modifications disrupt natural ecosystems and alter the spatial dynamics among vectors, wildlife hosts, and humans [12]. Forest fragmentation creates extensive edge habitats that are increasingly exploited by forest-dwelling *Anopheles* mosquitoes of the Leucosphyrus Group, thereby facilitating more human encounters in these degraded zones. At the same time, macaque hosts are driven into these areas in search of food and shelter, also bringing them into closer proximity with human populations [12]. This ecological convergence heightens the likelihood of zoonotic spillover, thereby amplifying transmission risk. Consequently, most *P. knowlesi* and other zoonotic malaria cases are reported among individuals whose occupations or activities place them at the forest-human interface, including forestry workers, agricultural labourers, military personnel, and those engaged in recreational or subsistence forest activities [13]. The interplay among land-use change, vector and host ecology, and human behaviour therefore drives the current epidemiological patterns observed across Southeast Asia.

Currently, no specific or targeted interventions are available to control and eliminate zoonotic malaria, with *P. knowlesi* remaining a significant public health threat in Southeast Asia. One of the key strategies for breaking the transmission cycle of zoonotic malaria is vector control. However, existing tools, largely designed for human malaria, are often ineffective in settings where transmission involves non-human primate reservoirs. Conventional interventions such as insecticide-treated bed nets (ITNs) and indoor residual spraying (IRS) provide limited protection in these ecologies due to the predominantly outdoor and early-biting behaviours of vectors and the sylvatic nature of transmission [10].

Compounding these challenges is the complexity of the major vector complexes in Southeast Asia. Accurate species identification remains difficult because many members exhibit overlapping morphological characteristics. Indeed, accurate species identification is critical for distinguishing primary vectors from non-vectors. Without this precision, control efforts risk being misdirected toward species that do not drive sustained transmission, thereby undermining the efficacy and cost-effectiveness of interventions, particularly in complex ecological settings where sibling species exhibit divergent biting and resting behaviours [8]. Similar diagnostic limitations exist for zoonotic *Plasmodium* species, which closely resemble human malaria parasites under light microscopy, making accurate diagnosis highly dependent on skilled personnel [14]. While molecular tools like PCR provide definitive identification, their high cost often renders them inaccessible for routine diagnosis in many Southeast Asian countries. Consequently, they must rely on microscopy, which makes distinguishing parasites difficult. Accurate diagnosis remains essential because zoonotic malaria involves wildlife reservoirs and environmentally driven transmission dynamics that require integrated environmental and vector-control strategies, whereas non-zoonotic human malaria is primarily controlled by prompt treatment, vector control, and interruption of human-mosquito-human transmission. Furthermore, the ecological and environmental drivers that intensify cross-species transmission remain poorly understood, largely because of limited and fragmented data on the distribution, behaviour, and bionomics of the mosquito vectors. The scarcity of comprehensive entomological studies beyond a few well-documented regions, such as Malaysia and Thailand, hinders the development of evidence-based interventions in neighboring Southeast Asian countries that report equally high *P. knowlesi* burdens but remain critically understudied.

Thus, this review aims to highlight current knowledge of zoonotic malaria and the complexities of its vectors within a rapidly changing landscape. By emphasising the taxonomic, ecological, and operational challenges, we identify critical gaps that impede effective vector control and outline opportunities to advance strategies tailored to zoonotic malaria transmission.

## Vector complexities: ecology and behaviour

### Primary vectors of zoonotic malaria

**Fig. 1:**
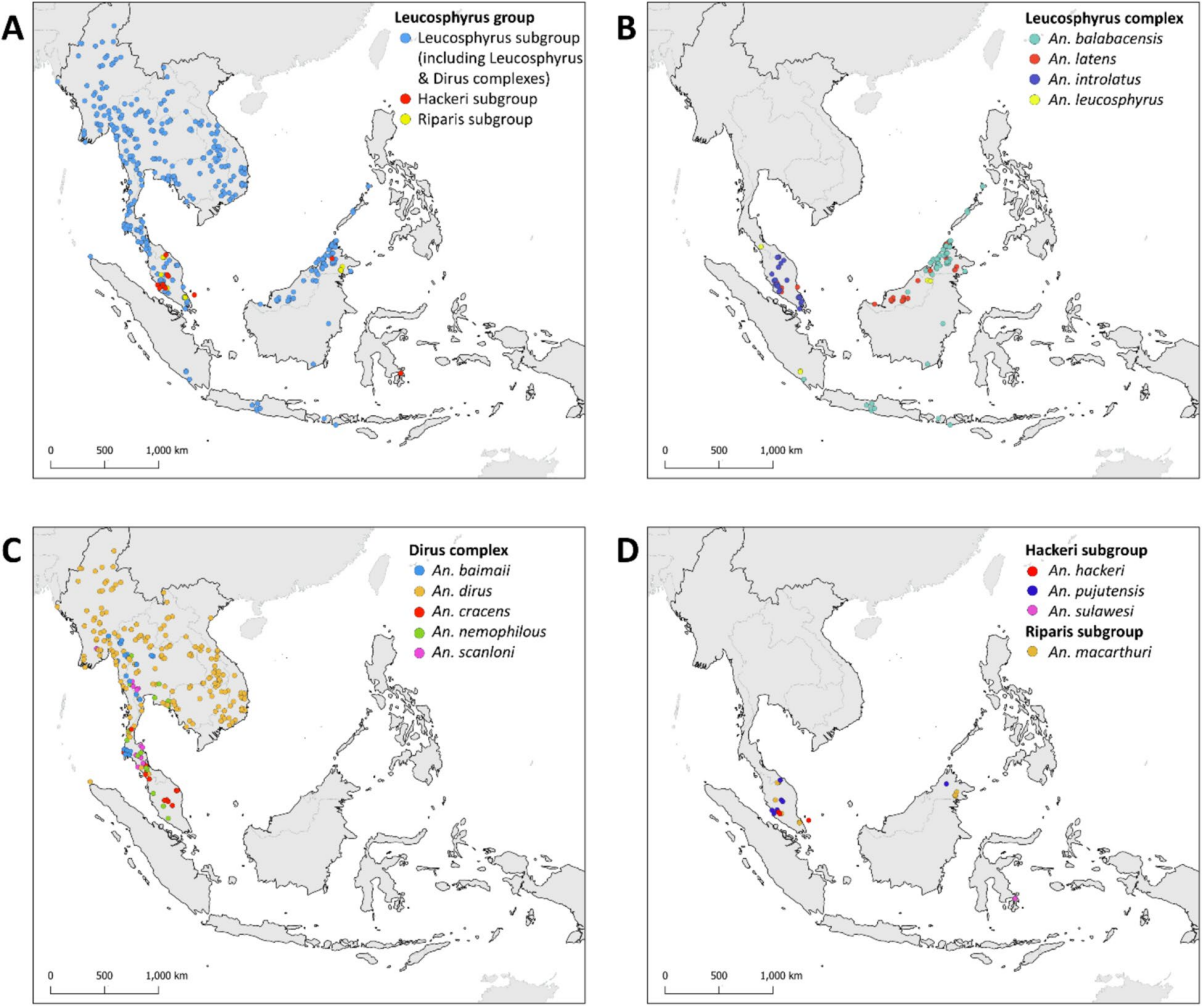
Distribution of reported occurrence of Leucosphyrus Group mosquitoes from 1957 to 2023 stratified by subgroups (**A**), Leucosphyrus complex species (**B**), Dirus complex species (**C**), and Hackeri and Riparis subgroup species (**D**)

*Anopheles* species from the Leucosphyrus Group are among the primary mosquito vectors for zoonotic malaria transmission in Southeast Asia, particularly in forested areas [11]. However, their species composition and geographic distribution vary considerably across Southeast Asia (Fig. 1). These distributions were mapped using a comprehensive dataset synthesized from peer-reviewed literature and historical records (Additional file 1: Table S1). The *Anopheles* Leucosphyrus Group comprises three subgroups: Leucosphyrus, Hackeri, and Riparis. Within the Leucosphyrus subgroup, there are two complexes: the Leucosphyrus and the Dirus. The *Anopheles dirus* sensu lato (Dirus complex) comprises several morphologically similar sibling species, including *Anopheles dirus* sensu stricto, *An. baimaii*, *An. scanloni*, *An. nemophilous*, *An. takasagoensis*, *An. elegans*, and *An. cracens*. Although they are morphologically very similar, they differ markedly in their behaviours and vectorial capacities [15–17]. This scenario is also similar for the Leucosphyrus complex, which comprises *Anopheles leucosphyrus*, *An. latens*, *An. introlatus*, *An. balabacensis*, and *An. baisasi*. Indeed, several members of both complexes, notably *An. dirus*, *An. cracens, An. latens, An. introlatus*, and *An. balabacensis*, play significant roles as vectors of knowlesi malaria across Southeast Asia [18, 19]. These mosquitoes exhibit flexible host-feeding behaviour, feeding on both humans and macaques in forested environments, enabling their role as a bridge vector in zoonotic malaria transmission [20]. The simio-anthropophagic behaviour of *Anopheles* mosquitoes from the Leucosphyrus Group was previously studied, especially for *An. introlatus* (Leucosphyrus Complex) and *An. cracens* (Dirus Complex) [21]. This opportunistic feeding behaviour facilitates spillover between sylvatic and human transmission cycles, particularly in forest and forest-fringe settings. Additionally, forest ecology supports persistent vector populations by providing suitable oviposition sites and abundant hosts [19]. Most *Anopheles* from the Leucosphyrus Group exhibit exophilic behaviour, resting outdoors after feeding, and can display early evening biting activity in some populations [22–24]. These behavioural traits influence exposure risk and make the indoor vector control measures ineffective. Human activities in forested areas, including agriculture and logging, increase contact between these vectors and humans, thereby sustaining high transmission levels, often referred to as “forest malaria” [19, 25]. The distribution of *Anopheles* from the Leucosphyrus Group is generally linked to dense forest zones across Southeast Asia, with varying vector prevalence depending on environmental changes and land use [5, 17, 19]. The efficiency of these species as malaria vectors is also related to their high susceptibility to malaria parasites and their high survival rates, which are critical for parasite development and transmission [23].

### Outdoor, forest-associated biting patterns

*Anopheles* mosquitoes from the Leucosphyrus Group exhibit predominantly outdoor biting behaviour [26, 27]. These vectors commonly bite humans outdoors in forested areas, particularly where human activities such as logging, agriculture, and rubber tapping occur [17, 28]. Besides, evidence indicates that several vector species exhibit opportunistic biting in peri-domestic and village settings, including around human dwellings, highlighting a potential risk of transmission beyond strictly forested environments [27, 28]. Thus, standard interventions such as ITNs and IRS that target indoor biting mosquitoes are less effective in zoonotic malaria settings [30]. *Anopheles* mosquitoes of the Leucosphyrus Group typically exhibit crepuscular to nocturnal biting behaviour, with activity beginning shortly after dusk and peaking between 18:00 and 21:00 h, depending on the mosquito species [12]. For example, *An. latens* in Sarawak and *An. balabacensis* in Sabah, Malaysian Borneo, showed a slightly earlier peak biting time around 18:00 and 19:00 h [24, 31], while mosquitoes from Peninsular Malaysia, such as *An. latens* and *An. cracens*, peaked slightly later, around 19:00 and 20:00 h, followed by *An. introlatus*, which peaked between 20:00 and 21:00 h [23]. Interestingly, some mosquito species, such as *An. dirus* in mainland Southeast Asia, exhibit dual peak biting time (20:00–22:00 h and 00:00–02:00 h), posing a risk to individuals engaged in forest-related activities during these periods [32]. However, peak biting times have often been generalised from limited, site-specific studies and require further investigation. While biting rhythms are largely species-specific and influenced by geographic location, the timing and intensity of host-vector contact can also be influenced by host behaviour, such as macaques’ movement and resting patterns, thereby shaping zoonotic malaria transmission dynamics [2].

In addition to peak biting times, ecotypes also influence mosquito species composition and biting behaviour. *Anopheles latens*, which was found to be responsible for transmitting *P. knowlesi* infection in Kapit, Sarawak, has been reported to bite humans in farms and forest fringes [31]. *Anopheles balabacensis*, the main vector of *P. knowlesi* in Sabah, is primarily found in large numbers near plantations, forest edges, and villages [33]. In addition to species differences, biting patterns are also influenced by seasonality and forest microclimatic conditions. In particular, increased rainfall and higher humidity generally enhance larval habitat availability and adult survival, leading to higher vector density and biting activity, whereas extreme rainfall may temporarily reduce activity by flushing breeding sites. Temperature also plays a key role, with moderate temperatures promoting mosquito development and host-seeking behaviour, while excessively high or low temperatures can suppress biting activity [12]. In some parts of Vietnam, *An. dirus* feeds on humans and macaques at forest edges and in forests [32, 34]. The outdoor biting behaviour also increases malaria risk for people engaged in nighttime forest activities [30, 35, 36]. This behaviour highlights the need for outdoor surveillance to better understand transmission and show the importance of targeted personal protection, addressing setting-specific dynamics and vector control needs.

### Seasonality and behavioural adaptation of *Anopheles* mosquitoes to a disturbed landscape

The abundance of *Anopheles* species is closely linked to seasonal rainfall patterns, with populations peaking during the monsoon season, and declines during drier months, reflecting strong seasonal dynamics. This increase is driven by the formation of additional breeding sites, such as puddles, ground pools, and water pockets, for some *Anopheles* vectors in forested and semi-disturbed habitats [36]. This seasonality directly influences malaria transmission intensity, with higher transmission risk coinciding with increases in vector population density and biting rate [12]. The persistence and increased abundance of these vectors in disturbed landscapes likely reflect increased habitat availability and altered microclimatic conditions, with possible contributions from the mosquitoes' behavioural plasticity, thereby sustaining transmission risk beyond peak monsoon periods [37]. The anthropogenic landscape change ultimately brings forest-dwelling *Anopheles* mosquitoes and macaques into close contact with humans, increasing the risk of disease transmission [38]. Indeed, *Anopheles* mosquitoes from the Leucosphyrus Group exhibit considerable ecological adaptability to fragmented or disturbed landscapes, including rubber plantations and secondary forests [17, 30, 39, 40]. For example, a study in Kudat, Malaysia, showed that the *An. balabacensis* is primarily found in large numbers near plantations, forest edges, and villages [33]. Landscape disturbance may create new or more favourable breeding sites, thereby enhancing vector survival and supporting sustained population growth [12, 41]. Thus, in addition to the increased availability of suitable breeding sites for *Anopheles* mosquitoes, the ability of these vectors to exploit a wide range of habitats highlights the ecological complexity of zoonotic malaria and underscores the need for integrated vector management strategies tailored to local environmental and socio-ecological contexts.

In addition, dynamic changes in mosquito behaviour are among the factors contributing to vector complexity in zoonotic malaria transmission. Observed variations in biting time, host preference, resting behaviour, and habitat use are likely driven by environmental change, land-use modification, and vector control pressure, reflecting context-dependent behavioural plasticity and ecological opportunism that influence contact patterns among vectors, wildlife reservoirs, and human populations. [42, 43]. Thus, understanding these behavioural adaptations is essential for interpreting transmission risk and for designing effective surveillance and control strategies in zoonotic malaria settings.

Nonetheless, adaptive behaviours, such as altered feeding times, increased outdoor biting (exophagic), and resting behaviour outside human settlements (exophilic) have been increasingly observed in primary malaria vectors, including in *An. dirus* complex species [19, 25, 44]. The behaviour changes have reduced the effectiveness of indoor-focused malaria control strategies, such as ITNs and IRS. Some mosquito populations also exhibit an adaptation to feed earlier in the evening or later in the morning when humans are less protected [45, 46]. These behavioural adaptations can be driven by selective pressures imposed by widespread insecticide use, habitat modification, and the patterns of human activity [19]. These behaviours demonstrate the importance of adaptive vector management strategies for sustaining transmission reduction. The ecological and genetic adaptations of *Anopheles* vectors to environmental changes should also be the primary focus of future research.

## Challenges in vector identification and surveillance

### Morphological identification difficulties

Distinguishing certain members of the *Anopheles* Leucosphyrus Group from other morphologically similar *Anopheles* species remains a major taxonomic challenge. Nevertheless, accurate identification of mosquito species is essential because different species vary in their capacity to transmit pathogens, depending on host preferences and biting and resting behaviours [47]. This challenge is particularly evident given the high morphological similarity among sibling species within *Anopheles* species complexes (Table 1). For instance, members of the *An. dirus* complex exhibit overlapping diagnostic characters, making them extremely difficult to distinguish taxonomically [48]. Such cryptic diversity can lead to misidentification, resulting in inaccurate assessments of species distribution, ecological traits, and vectorial capacity, ultimately affecting surveillance and targeted vector control strategies. For example, six morphologically cryptic species within the Leucosphyrus Group were reclassified, exhibiting extensive overlap in diagnostic characters such as wing scaling, leg banding, and adult external morphology, thereby rendering reliable identification using conventional morphological keys extremely difficult [16]. This exemplifies how morphological uniformity within sibling species complexes can mask true species diversity. This underscores the necessity of integrative taxonomy, combining detailed morphological examination with molecular and biogeographical evidence, to accurately delimit species. Such taxonomic ambiguity has direct implications for vector studies, as different cryptic species within the Leucosphyrus Group may vary in ecology and vectorial importance, leading to misinterpretation of transmission dynamics if misidentified [49–51], e.g. a study in Vietnam in which *Anopheles varuna*, a species not implicated in transmission, was incorrectly identified as *An. minimus* [52]. This misclassification resulted in misguided vector control efforts and the unnecessary allocation of limited resources. On the other hand, members of the *An. dirus* complex were historically grouped under *An. balabacensis* sensu lato, leading to taxonomic confusion. Subsequent resolution into sibling species (e.g. *An. baimaii*, *An. cracens*) revealed differences in distribution and vectorial importance, highlighting how earlier misidentifications obscured species-specific roles in malaria transmission [53].

**Table 1:** Taxonomic complexity among the zoonotic malaria vector complexes

Species name/complex	Key morphological characters	Overlapping features with other species	Possibility for misidentifications	References
*Anopheles dirus* *Anopheles takasagoensis* *Anopheles* affinis *takasagoensis*	Presector dark spot on vein R extending beyond presector dark (PSD), pale fringe spot between 1A and Cu2, accessory sector pale spot on subcosta	Morphological similarity between *An. dirus*, *An. takasagoensis*, and *An*. aff. *takasagoensis*; wing-spot patterns vary with geography and environment	High risk of misidentification; cryptic species indistinguishable morphologically; molecular/cytological confirmation needed	[54]
*Anopheles latens*, *Anopheles cracens*, *Anopheles scanloni*, *Anopheles baimaii*,	(a) PSD spot of vein R extending basally onto the level of humeral dark (HD) spot of costa, or beyond the middle of HD; apical pale band on palpomere 5 distinctly cream-colored or yellowish (*An. latens*) (b) Abdominal sternum VI with a small posteromedial patch of dark scales (*An. cracens*) (c) Pale scales on anterior veins of wing, white, contrasting with pale spots on posterior veins; middle dark area of foretarsomere 2 always long, usually without pale spots on dorsal surface, occasionally with 1–3 tiny separate pale spots (*An. scanloni*) (d) Presector dark (PSD) spot on vein R usually at level of PSD spot on costa, usually no more than middle of presector pale (PSP) spot of costa; foretarsomere 2 often with pale bands and pale spots fused and completely pale dorsally, or spots and bands longer, reducing dark area to narrower median band (*An. baimaii*) (e) proboscis longer than forefemur (ratio 1.19–1.31) and longer than maxillary palpus (palpus/proboscis 0.84–0.93); proboscis entirely dark-scaled; vein R PSD spot without pale breaks and PSD-PD spots with two pale interruptions; palpomere 5 apical/basal band ratio 0.83–2.67; hindtibia with apical pale band and a continuous dark longitudinal stripe extending basally (*An. mirans*)	Overlap within Leucosphyrus Subgroup (large white band on tibio-tarsal joint of hindleg; proboscis without pale scales on apical half) and hackeri Subgroup; redescribed and reclassification due to morphological similarity	Previous misidentification (a) *An. latens* as *An. leucosphyrus* A (b) *An. cracens* as *An. dirus* B (c) *An. scanloni* as *An. dirus* C (d) *An. baimaii* as *An. dirus* D (e) *An. mirans* as *An. elegans*	[48, 55]
*Anopheles dirus* complex	Presence of distinctive pale and dark banding patterns on the wings, particularly involving the costal and subcostal veins. However, these characters overlap extensively among sibling species, making reliable identification based on morphology alone difficult Molecular techniques are needed to differentiate members in the complex group	Sibling species with unclear boundaries; limited diagnostic information	Historical confusion and the need for re-evaluation of this complex group	[56–58]

The problem is also further compounded by a severe shortage of trained medical entomologists and taxonomists in the region. Expertise in morphological identification is highly specialised and requires years of training and regular practice. After the retirement of senior experts, the knowledge gap becomes wider, leaving many surveillance programmes dependent on a small number of specialists. This scarcity creates bottlenecks in routine vector monitoring and during rapid outbreak investigations [59, 60]. Interestingly, an external quality assessment of 19 laboratories demonstrated substantial variability in mosquito identification accuracy, with only 64% correct at the species level, highlighting significant limitations of morphology-based identification and the need for improved taxonomic capacity and quality control [61]. In addition, accurate taxonomic identification of mosquitoes is further complicated by the condition and quality of the collected specimens. Collection methods, such as CDC light traps, can physically damage mosquitoes, particularly through contact with the trap fan, leading to the loss of key morphological characters (e.g., wing or leg scales) required for reliable identification [62]. Consequently, damaged specimens increase the likelihood of misidentification and further challenge morphology-based taxonomic resolution.

As a result, there is an increasing reliance on molecular approaches such as DNA barcoding, PCR-based assays, and next-generation sequencing. These methods have become invaluable for distinguishing morphologically similar species and confirming vector incrimination. For example, molecular confirmation has been crucial in distinguishing *An. latens* from *An. introlatus* in Sarawak, Malaysia [63]. This example further highlights the limitations of morphology-based identification and emphasises the essential role of molecular tools in accurate vector incrimination and targeted control measures. Besides, barcoding using the COI gene has also uncovered hidden diversity in regional vector populations [64], revealing cryptic species complexes that would otherwise have remained undetected through morphological identification alone and further refining our understanding of vector-parasite relationships.

Despite their advantages, molecular tools have important limitations, as they depend heavily on the accuracy and completeness of reference sequences in public databases such as GenBank and BOLD [65]. Recent initiatives, such as the ANOSPP project, highlight ongoing efforts to develop curated, expertly validated reference datasets to improve the reliability of species identification [66]. Although currently focused on African mosquitoes, its broader goal of establishing a comprehensive reference index for the entire *Anopheles* genus makes it highly relevant for species identification, including zoonotic malaria vectors in Southeast Asia. Another limitation of molecular identification is that such facilities are not always readily available in remote areas, where challenges related to sample storage and transport can further constrain its application, leading to reliance on outdated morphological keys [67]. In general, mosquito identification can be performed using taxonomical identification and further confirmed using molecular methods; however, each approach has its own advantages and limitations (Table 2).

**Table 2:** Methods to identify vectors

Method	Description	Advantages	Disadvantages	References
Morphological identification (taxonomy)	Identification based on mosquito physical traits and structures under microscopy	Simple, low cost, widely used; effective for well-known species	Requires expert entomologists; prone to technical and human errors; cannot distinguish cryptic species accurately	[68]
Geometric morphometrics	Modern morphometric measurement techniques, developed from measurement standards, rely on geometric principles to assess size and shape variables for species identification in the study of morphological variation	Inexpensive, rapid, and does not require sophisticated equipment	The technique should be revalidated for each new target area to avoid misclassification caused by environmentally driven morphological variation in the mosquito population; large-sample studies are needed to strengthen confidence in its reliability	[58]
Crossbreeding experiments	Testing reproductive compatibility between mosquito populations to delineate species boundaries	Provides biological species confirmation; clarifies species complexes	Time-consuming, labor-intensive; impractical for large-scale field studies	[69]
Cytogenetic methods	Analysis of chromosomal banding patterns and karyotypes for species identification	Useful for distinguishing closely related species; provides genetic evidence	Requires specialized laboratory facilities and expertise; low throughput	[70]
DNA probe techniques	Hybridisation of labelled DNA probes to identify species-specific genetic sequences	High specificity; useful for detecting cryptic species	Use of radioactive probes (traditional) raises safety issues	[71]
Non-radioactive DNA probes	Safer alternative to radioactive probes using fluorescent or chemiluminescent labels	Safer and more environmentally friendly; comparable specificity to radioactive probes	Still slower and less sensitive compared to PCR-based methods; it requires specialized detection	[72]
Multiplex PCR	PCR variant amplifying multiple targets in one reaction to identify several species simultaneously	Efficient, cost-effective; saves time and reagents by combining assays	Optimization can be complex; there is a risk of primer interactions leading to false negatives or positives	[73]
qPCR and RT-qPCR	Quantitative and reverse transcription PCR measuring DNA/RNA levels to detect parasites and vectors	Quantitative data; very sensitive; can distinguish active infections by detecting RNA	High-cost equipment requires technical expertise and careful sample handling	[74]
Nested PCR	Two-step PCR to increase specificity and sensitivity for detecting low parasite loads	Improves detection of rare infections; reduces false positives	Increased contamination risk due to multiple steps	[75]
Next Generation Sequencing	High-throughput DNA sequencing to analyze whole genomes or gene regions	Comprehensive, fast, low DNA requirement, and detects wide genetic variations	Costly setup, requires bioinformatics expertise, prone to PCR bias, and prone to sequencing errors	[68]
Mitochondrial DNA Barcoding	Sequencing of mitochondrial genes to discriminate species and detect cryptic diversity	High resolution; useful for species complexes; reproducible globally	Sequencing cost; requires reference databases and molecular labs	[76, 77]
Microsatellites and amplified fragment length polymorphisms (AFLPs)	Highly polymorphic DNA markers for population genetics and species differentiation	Useful for fine-scale studies of gene flow and species boundaries	Requires specialised labs; sample quality is important; data analysis can be complex	[78]

### Lack of comprehensive entomological studies

Over the decades, there has been a noticeable decline in the use of classical entomological approaches, such as dissection-based studies, for malaria vectors. Indeed, dissections of the salivary glands and midguts are considered the gold standard for vector incrimination, as they allow direct detection of natural sporozoite and oocyst infections, thereby providing definitive confirmation of vector status [79]. However, with the advent of molecular methods, these labour-intensive techniques have gradually been replaced. This shift has reduced opportunities for training and field capacity in morphological and anatomical investigations [80, 81].

Classical entomological indices such as parity rate, sporozoite detection, and age-grading remain critical for vector incrimination and for estimating the proportion of mosquitoes that survive long enough to transmit malaria. Although newer technologies, such as near-infrared spectroscopy, are available for mosquito age grading, they have limitations, including dependence on robust calibration datasets, limited transferability across settings, and the inability to provide direct biological information (e.g., parity, ovarian development, and infection status) [82]. Moreover, this method has not yet been validated in zoonotic malaria vector species. On the other hand, the classic parity dissections provide essential information on vector longevity and reproductive cycles, while sporozoite rates give direct evidence of infectivity [79]. Yet, these measures are rarely incorporated in contemporary field studies, leading to an incomplete picture of local transmission dynamics.

These classical metrics are indispensable for calculating entomological inoculation rate (EIR) and vectorial capacity, which integrate mosquito density, human biting rates, survival, and sporozoite prevalence. Without reliable estimates of mosquito longevity and infection rates, models of malaria transmission risk are likely to be misleading or incomplete. This challenge is further compounded by the limited availability of empirical data, requiring models to rely on assumptions that may not adequately capture the complexity and diversity of vector communities. This is particularly concerning for zoonotic malaria in Southeast Asia, where the role of emerging vectors remains poorly defined in some of the countries [24, 81, 83].

The decline in the use of dissection-based approaches is partly attributable to their labour-intensive nature and the high level of technical skill required, particularly for dissecting mosquito salivary glands. Salivary gland dissection can be particularly challenging for certain mosquito species due to the glands' small size and fragility. These challenges are further amplified in entomological studies conducted in areas with high vector abundance, where large numbers of specimens must be processed within a limited time frame, and skilled personnel are often in short supply. The difficulty is exacerbated when dissections must be performed on live mosquitoes, as dead specimens tend to desiccate rapidly, become fragile, and are more prone to tissue damage, thereby complicating gland extraction [79]. Nevertheless, these limitations can be addressed through targeted capacity building, including training and upskilling of field staff in mosquito dissection techniques, as well as strategic planning to optimise workflows. Despite these challenges, dissection remains a critical method for vector incrimination, as it provides definitive evidence of infectivity by detecting sporozoites in the salivary glands.

## Challenges in controlling zoonotic malaria

### Limitations of current control strategies

Conventional malaria control strategies, such as ITNs and IRS, were developed to protect against indoor-biting and indoor-resting mosquito species. While these measures have proven highly effective against human malaria parasites [84, 85], they offer limited protection against zoonotic malaria parasite transmission. In zoonotic malaria transmission, key vector species such as *An. latens*, *An. balabacensis*, and *An. dirus* predominantly bite outdoors and during the early evening [24, 31, 44], when people are still active outside their homes. This behavioural mismatch substantially reduces the effectiveness of ITNs and IRS. For example, studies conducted in Sabah, Malaysia, have demonstrated that *An. balabacensis*, the primary vector of *P. knowlesi*, exhibits predominantly early outdoor biting behaviour, with a considerable proportion of human biting occurring before 21:00 h. [24, 86]. This early evening activity occurs before most individuals retire indoors or seek protection under bed nets, thereby limiting the protective impact of ITNs and IRS against zoonotic malaria transmission. As a result, even in areas with high ITNs coverage, zoonotic malaria parasite transmission persists, underscoring the inadequacy of current indoor-focused tools for controlling zoonotic malaria [87].

The lack of effective outdoor mosquito control tools represents one of the greatest obstacles in zoonotic malaria elimination. Methods such as spatial repellents [88], insecticide-treated clothing [89], attractive toxic sugar baits [90], and larval source management [91] have been explored but have not yet been studied for the vectors of zoonotic malaria in Southeast Asia. Forest-related activities and occupational exposure, such as farming, logging, or military activities, further complicate implementation. Unlike human malaria control, outdoor-focused interventions for zoonotic malaria lack standardized guidelines, robust large-scale trial data, and sustainable long-term models. Without the development and implementation of targeted interventions against outdoor-biting vectors, zoonotic malaria will continue to pose a significant challenge to current malaria control and elimination efforts.

Surveillance efforts in zoonotic malaria-endemic regions primarily concentrate on tracking human cases. Established methods include Thailand’s 1-3-7 surveillance strategy [92] and surveillance systems incorporating active case detection, passive case detection, and mass blood survey practiced in several Asian-Pacific countries [93]. Although these methods effectively reduce the clinical burden of malaria, they often overlook monitoring of vectors and primate reservoirs, creating critical gaps in understanding zoonotic malaria transmission. Tracking *Plasmodium* infections in primate populations presents logistical and ethical challenges, requiring expert teams and collaboration with wildlife authorities. Similarly, entomological surveillance is often limited to small-scale studies; thus, it lacks continuous data on vector populations, biting patterns, and infection rates. Additionally, entomological work presents risks, such as exposure of sampling personnel to vector bites [94–96]. Entomological surveillance often involves reactive mosquito surveillance, focusing efforts in response to malaria clusters. This approach can be operationally complex due to the need for quick deployment, coordination, and adaptation to diverse environments. Importantly, without integrated surveillance across humans, vectors, and primate hosts, policymakers lack the comprehensive data necessary to design targeted interventions, although spatial modelling can help to some degree. This surveillance gap perpetuates reactive rather than proactive responses, allowing zoonotic malaria to remain a hidden and under-reported public health threat.

### Limitations of malaria surveillance in wildlife reservoirs

A fundamental distinction between zoonotic and human malaria lies in the presence of wildlife reservoirs, specifically non-human primates (NHPs). Unfortunately, the eradication strategies cannot feasibly or ethically target these reservoir hosts because of ecological, conservation, and ethical constraints. Unlike humans, macaque populations cannot be systematically treated with antimalarial drugs or deliberately displaced from their natural habitats without significant logistical challenges and potential ecological consequences, which may inadvertently increase human-vector contact. While wildlife-targeted interventions work for zoonoses like rabies, treating macaques is hindered by the need for sustained drug levels, the risk of driving antimalarial resistance, and the behavioural ecology of primates, whose complex foraging and social structures make consistent baiting challenging [97]. Furthermore, the overlapping habitats of NHPs, humans, and competent *Anopheles* vectors create transmission interfaces that facilitate cross-species parasite exchange. As human settlements expand into forested landscapes and agricultural frontiers, the frequency and intensity of human-primate-vector contact increase, heightening the risk of zoonotic malaria spill over to human populations [38, 98]. This intricate interplay of ecological, behavioural, and entomological factors underscores why zoonotic malaria may persist even when conventional human-focused control measures successfully reduce or interrupt human malaria parasite transmission.

A further limitation is that surveillance of *Plasmodium* in wildlife reservoirs is inherently sparse, biased, and difficult to standardize; thus, estimation of reservoir infection is often uncertain. In Southeast Asia, macaque surveillance often relies on opportunistic or convenience sampling, which can bias prevalence estimates and limit extrapolation to broader populations. Moreover, even when molecular methods are applied, variability in sample quality and preservation can reduce detection sensitivity and influence apparent prevalence [99]. Besides, surveillance of wildlife reservoirs is rarely aligned spatially and temporally with mosquito and human sampling, making it difficult to relate macaque infection dynamics to vector abundance and human case patterns. Although recent integrated analyses highlight the value of coordinated, multi-host surveillance, such approaches remain uncommon and methodologically challenging to implement in most Southeast Asian countries [100]. This is consistent with recent systematic reviews highlighting insufficient sampling efforts, uneven geographic and host coverage, and publication bias in wildlife surveillance, all of which collectively constrain risk mapping and the design of targeted interventions at the human-primate interface [101].

## Pathways forward: towards tailored strategies

The first step in strengthening zoonotic malaria research is to revitalize entomological capacity across Southeast Asia. Many vector control programmes now rely almost exclusively on molecular data, while traditional entomological skills such as mosquito collection, identification, and dissection are gradually disappearing [80, 102]. Sustained investment in laboratories, insectaries, and field surveillance units will be critical to ensure that future research can address emerging transmission challenges with both classical and modern approaches. However, where resources are limited, alternative approaches such as regional collaboration, shared infrastructure, and cost-effective, field-adapted tools can help support integrated research and surveillance. Besides, a long-term solution must include training programmes for capacity building that emphasise taxonomy and classical entomological methods. The shortage of trained mosquito taxonomists has created a serious knowledge gap in the region. Programmes that reintroduce practical modules on morphological identification, mosquito dissections, and mosquito age-grading can ensure continuity of expertise [59, 103]. Training should also be embedded in postgraduate curricula, national malaria programmes, and regional workshops to rebuild a skilled workforce.

The integration of morphological identification with molecular tools represents the most robust strategy in malaria entomology, particularly for accurate confirmation of mosquito species. Morphological identification through mosquito taxonomy provides quick, low-cost field-based identification, while molecular diagnostics, including DNA barcoding and sequencing, help resolve cryptic species complexes and confirm vector species [51, 65]. Thus, building curated molecular databases such as the ANOSPP project, VectorBase, and malariaGEN, which are linked to voucher specimens, will reduce the risk of misidentification and ensure reliable species assignments. Regional initiatives to share curated reference material will strengthen molecular taxonomy across Southeast Asia. Systematic mapping using both morphology and molecular approaches will help refine risk models and guide targeted control measures [50]. Such efforts will also identify priority zones where transmission may be driven by neglected or newly emerging vector species. Indeed, vector incrimination remains the cornerstone of malaria transmission studies. Renewed efforts are needed to quantify parity rates, sporozoite infections, and biting behaviours through dissections, feeding assays, and molecular confirmation. These data underpin calculations of entomological inoculation rates and vectorial capacity, both of which are essential for understanding transmission intensity [81, 83]. Expanding such studies across multiple ecological zones will clarify the role of less-studied vectors.

On the other hand, traditional vector control tools alone may be insufficient in forest and forest-fringe transmission settings, highlighting the need for complementary and context-specific control strategies. Novel approaches such as spatial repellents, attractive toxic sugar baits (ATSBs), and gene-drive technologies offer new opportunities [92–106]. The general concept of spatial repellency, such as transfluthrin, is to prevent vectors from entering spaces occupied by potential human hosts, thereby reducing encounters between humans and vectors and eliminating or reducing the risk of pathogen transmission to humans [88, 104]. In terms of genetic manipulation, gene drive systems hold considerable promise for future vector control by biasing inheritance through DNA cleavage and repair or by spreading disease-refractory genes within mosquito populations, thereby reducing vector competence and potentially suppressing mosquito populations [105]. On the other hand, the ATSB solution utilizes insecticide-treated food baits to target adult mosquitoes [106]. While this method showed promise in Mali's arid climate [107], a large-scale trial failed to reduce malaria incidence, likely due to implementation challenges [90]. In the context of Southeast Asian forests, the efficacy of ATSBs is further questioned by the sheer density of competing natural floral sugar sources, which may lure vectors away from the baits. Additionally, unlike controlled urban environments, the deployment of ATSBs in biodiverse forest ecosystems carries a high risk of off-target impacts on beneficial pollinators and non-vector species. Consequently, while these approaches exist, their suitability for zoonotic malaria control in Southeast Asia remains highly speculative and requires rigorous ecological safety assessments.

Indeed, vector control measures must be continually tailored to changing landscapes, as environmental modification alters mosquito ecology and drives shifts in mosquito behaviour, which directly influences the malaria transmission dynamics. Land-use modification, deforestation, and climate variability influence mosquito distribution, macaque habitats, and human exposure [12]. Effective control must incorporate habitat-based management, such as establishing protected zones to minimize human-macaque overlap and tightening regulations on logging and forest-edge settlements. Besides, an advanced modelling approach that integrates ecological, climatic, and demographic data will provide critical foresight into future zoonotic malaria risks [108, 109]. These models can help prioritize interventions and resource allocation. In addition, improving zoonotic malaria surveillance requires expanding current efforts through innovation and research, particularly by leveraging advances in technology and the application of artificial intelligence (AI). Technologies such as aerial imaging, geo-tracking, and bioacoustics provide new methods for monitoring macaques, particularly in dense forests or remote locations [110, 111]. For vector surveillance, a novel approach to canopy-level sampling could enable the detection of uncommon vector species and shed light on lesser-known transmission pathways in the forest canopy [112]. In addition, incorporating artificial intelligence into imaging can enhance detection of malaria parasites, oocysts, and sporozoites, as well as vector identification, complementing routine microscopy and boosting accuracy [113, 114].

Across Southeast Asia, numerous malaria control measures have been implemented; however, most are designed to reduce human malaria, and only a limited number address outdoor-biting vectors implicated in zoonotic transmission (Table 3). Over the long term, national elimination strategies should incorporate zoonotic malaria components, as current programmes largely prioritise human-restricted *Plasmodium* species such as *Plasmodium falciparum* and *Plasmodium vivax*, leaving zoonotic malaria insufficiently addressed [115]. Incorporating zoonotic malaria surveillance and control within malaria elimination frameworks helps prevent programmatic gaps and sustain elimination gains, especially in settings where declining human malaria incidence may mask ongoing transmission from zoonotic reservoirs. Zoonotic malaria control demands cross-sectoral policies based on a One Health framework, enabling coordinated surveillance, prevention, and response across human health, wildlife, and environmental management [12]. Understanding how human activities, behaviours, and lifestyles impact ecosystems is essential for interpreting disease dynamics and informing public policies. The One Health concept emphasises the integration of human, animal, plant, ecosystem, and biodiversity health [38, 116]. By integrating ecological, epidemiological, and social interventions, One Health approaches offer the most comprehensive and sustainable pathway forward.

**Table 3:** Existing and conceptual interventions in combating zoonotic malaria

Category	Technique	Description	Existing or conceptual interventions targeting zoonotic malaria	References
Surveillance and diagnostics	Incorporating zoonotic malaria into national malaria surveillance, management, and treatment policy	Recognizing and formally integrating zoonotic malaria, primarily *Plasmodium knowlesi*, into existing national malaria surveillance, management, and treatment systems. This includes adapting surveillance methods such as active case detection, passive case detection, mass blood surveys where appropriate, molecular confirmation, and updated treatment protocols. At present, *P. knowlesi* are routinely reported and managed as part of national malaria programme in Malaysia, Thailand, Cambodia, Indonesia, and Brunei	Existing	[1, 117]
Surveillance and diagnostics	Proactive surveillance for detecting symptomatic/asymptomatic infections in high-risk communities	Active screening of communities living in malaria-risk areas for early detection of infection, including asymptomatic cases, to prevent disease progression and onward transmission. This approach enables timely treatment and strengthens malaria control efforts at the community level	Existing	[118, 119]
Surveillance and diagnostics	Implementing PCR diagnostic policy	Routine use of PCR-based methods for species confirmation of zoonotic malaria parasites to enhance diagnostic sensitivity and specificity, thereby reducing the risk of species misidentification, particularly in cases with low parasitaemia or morphologically similar *Plasmodium* species. This approach supports accurate case management, surveillance, and reporting of zoonotic malaria	Existing	[120]
Surveillance and diagnostics	Point-of-care diagnostics	Point-of-care diagnostics, such as rapid diagnostic test (RDT) kits, are essential for quick field screening of zoonotic malaria. Emerging tools, including haemozoin-based assays like the Gazelle device, offer a rapid, portable alternative that can enhance detection in low-resource and remote settings	Existing research outputs are available; however, they have not been widely adopted for routine use	[121]
Surveillance and diagnostics	Risk mapping and predictive modelling	Geospatial analysis of malaria transmission cases in association with environmental variations to identify high-risk zones and prioritise surveillance efforts	Existing	[5, 108, 109, 122]
Surveillance and diagnostics	Parasite biology research	Research into the zoonotic malaria life-cycle, genetics, drug resistance, and transmission to improve diagnostics, treatments, and control strategies. Such studies provide critical evidence to guide targeted interventions and inform policy decisions for effective zoonotic malaria management	Existing	[123]
Surveillance and diagnostics	Artificial intelligence (AI)-assisted diagnostics	Machine learning or AI tools for automated blood smear analysis or microscopy to detect and quantify parasites more accurately and quickly	Under development: promising research outputs available, but limited real-world adoption	[114]
Surveillance and diagnostics	Digital and mobile health reporting systems	Use of digital platforms and mobile applications for real-time malaria case reporting and surveillance data management. These tools can facilitate timely detection of zoonotic malaria cases, improve data integration across human, vector, and animal health sectors, and support rapid public health responses	Conceptual	[124]
Human-targeted	Community education	Raising awareness of zoonotic malaria exposure, promoting bite prevention, early treatment-seeking, and reducing human-vector-NHP contact	Existing	[125]
Human-targeted	Community participation in malaria control programmes	Community participation in malaria control programmes involves engaging local populations in case reporting, promoting preventive measures, and supporting entomological monitoring in remote or forested areas. Empowering communities through awareness, training, and collaboration enhances early detection, improves intervention uptake, and strengthens the sustainability of malaria control efforts, particularly for zoonotic malaria	Existing	[126–128]
Human-targeted	Chemoprevention	Preventive antimalarial drugs for individuals at risk of exposure to zoonotic malaria. Chemoprophylaxis may be considered for high-risk groups, such as forest workers or individuals entering endemic areas, to reduce the likelihood of *P. knowlesi* infection and associated severe disease	Existing as chemoprophylaxis against malaria in general but with limited evidence of effectiveness against zoonotic malaria	[120, 129]
Human-targeted	Vaccine	The development of vaccines to prevent zoonotic malaria transmission remains a long-term research priority. Although substantial research on *P. knowlesi* continues, there is currently no licensed *P. knowlesi* vaccine available. However, several studies are actively investigating potential vaccine candidates for their roles in inhibiting invasion and blocking transmission	Conceptual	[123]
Vector control	Outdoor residual spraying	Application of insecticides to outdoor vegetation or shelters to target exophilic vectors. However, the potential environmental and ecological impacts of such interventions require further investigation to ensure their safety, sustainability, and minimal effects on non-target organisms	Existing	[130, 131]
Vector control	Insecticide-impregnated clothing	Treating clothing with repellents or insecticides for forest workers provides personal protection against outdoor mosquito bites, particularly from exophagic and exophilic vectors. For example, permethrin-impregnated clothing has been evaluated as a supplementary personal protective measure to reduce mosquito landing and biting during forest-related activities	Existing for general vector control but with limited evidence for targeting zoonotic malaria vectors	[29, 132, 133]
Vector control	Spatial repellent	Devices releasing repellents to create bite-free zones around individuals or activity areas. Spatial emanators using volatile pyrethroids such as transfluthrin have been shown to reduce mosquito landing and biting and may be useful in outdoor or forest settings where conventional control measures are less effective	Existing for general vector control but with limited evidence for targeting zoonotic malaria vectors	[134, 135]
Vector control	Insecticide resistance monitoring	Insecticide resistance monitoring for vector control involves assessing resistance patterns in mosquito populations to optimize vector control strategies. Regular surveillance of resistance mechanisms and susceptibility profiles helps guide the selection and rotation of insecticides, thereby maintaining intervention effectiveness and delaying the development of further resistance	Existing for general vector control but with limited evidence for targeting zoonotic malaria vectors	[136]
Vector control	Larvivorous fish	Introduction of larvivorous fish species such as *Gambusia affinis* and *Poecilia reticulata* to consume mosquito larvae in identified breeding sites. However, for zoonotic malaria vectors, locating and accessing suitable breeding habitats can be challenging due to their dispersed, cryptic, and often forest-associated nature, which may limit the effectiveness of this approach	Conceptual	[137]
Vector control	Larval source management (Bacterial and chemical larvicides)	Larval source management using bacterial and chemical larvicides, such as pyriproxyfen, plays an important role in reducing mosquito populations at the immature stages. The use of larvicidal bacteria, including *Bacillus thuringiensis var. israelensis* and *Bacillus sphaericus*, effectively targets mosquito larvae in identified breeding habitats while minimizing impacts on non-target organisms, making these approaches suitable components of integrated vector management when breeding sites are accessible and well defined	Conceptual. Most studies involving *Anopheles* mosquitoes were conducted in Africa	[138, 139]
Vector control	Proactive surveillance to monitor vector distribution, bionomics, blood-meal sources, and parasite prevalence	Entomological surveillance is essential for identifying vector species, determining infection rates, and assessing transmission risk. These data provide critical evidence to guide targeted vector control interventions and improve understanding of zoonotic malaria transmission dynamics	Existing	[21, 23, 140, 141]
Reservoir and ecological control	Proactive surveillance for detecting malaria parasites in reservoirs	Sampling non-human primates (NHPs) for parasites to monitor reservoir infection rates and assess zoonotic potential. These data help clarify spillover risks to human populations and support the design of effective One Health surveillance and control strategies	Existing	[51, 142, 143]
Reservoir and ecological control	Reservoir distribution monitoring	Systematically tracking and mapping the geographic distribution, population density and movement patterns of NHPs	Existing	[144–146]
Reservoir and ecological control	Forest restoration	Replanting trees, rehabilitating degraded forests, restoring natural vegetation cover, and promoting sustainable land management in areas previously affected by deforestation, fragmentation, or conversion to agriculture or oil palm plantations. These efforts aim to rebuild forest ecosystems and reduce forest–edge interfaces where interactions among humans, non-human primates, and mosquito vectors are most intense	Conceptual	[147, 148]
Reservoir and ecological control	NHP behavioural modification (Push and pull tactics)	Push–pull strategies reduce non-human primate presence near human activity areas through deterrents or habitat modification (‘push’), while enhancing forest resources to attract primates away from settlements (‘pull’), thereby minimizing human–primate-vector contact	Conceptual	[120]
Reservoir and ecological control	Treatment using impregnated oral bait	Applying treated baits, oral contraceptives, or endectocides to non-human primates (NHPs) as a potential strategy to reduce reservoir infection. Such approaches aim to lower parasite prevalence or vector survival in NHP populations, but require careful evaluation of efficacy, safety, ethics, and ecological impacts before implementation	Conceptual	[120]

## Conclusions

Zoonotic malaria continues to pose a major hurdle to malaria elimination efforts in Southeast Asia. Its persistence is strongly driven by the interconnected challenges of human activities, vector ecology, and non-human primate reservoirs, making it a significant barrier even in areas where human-only malaria has been successfully reduced. Vector identification and surveillance remain major bottlenecks in malaria control, as misidentification and insufficient longitudinal monitoring can misdirect interventions and obscure an accurate understanding of the disease's transmission dynamics. These complexities underscore the need for strengthened entomological research and capacity building to support more accurate vector detection, behaviour profiling, and targeted control strategies. Enhancing surveillance systems, improving diagnostic accuracy, and implementing region-specific and ecology-specific interventions are essential for mitigating zoonotic transmission. The ecological and genetic adaptations of *Anopheles* vectors to environmental change should be a major research priority, while advancements in molecular diagnostics can facilitate earlier case detection and improve treatment outcomes. Future efforts should also explore innovative and sustainable vector management tailored to local ecologies. Importantly, integrated, One Health-oriented solutions that consider human, animal, and environmental interfaces should be prioritized to achieve long-term success. Collaboration among researchers, public health professionals, wildlife experts, and policymakers remains vital for designing and implementing effective elimination strategies. Strengthened cross-border activities and data-sharing frameworks will further enhance the region’s capacity to monitor zoonotic malaria and evaluate the impact of interventions. By combining robust scientific research, strengthened entomological capacity, and coordinated public health action, Southeast Asia may be better positioned to progress towards malaria elimination despite the unique challenges posed by zoonotic transmission.

## Supplementary Information

Additional file1.

## Data Availability

Data supporting the main conclusions of this study are included in the manuscript.
